# Symbolic violence experienced by frontline healthcare professionals during the COVID-19 pandemic

**DOI:** 10.1590/0034-7167-2024-0237

**Published:** 2025-04-28

**Authors:** Karla Gonçalves Camacho, Saint Clair dos Santos Gomes, Maria de Fátima Junqueira-Marinho, Adriana Teixeira Reis, Dimitri Marques Abramov, Daniella Campelo Batalha Cox Moore

**Affiliations:** I Instituto Nacional de Saúde da Mulher, da Criança e do Adolescente Fernandes Figueira, Fundação Oswaldo Cruz. Rio de Janeiro, Rio de Janeiro, Brazil; II Universidade do Estado do Rio de Janeiro. Rio de Janeiro, Rio de Janeiro, Brazil; III Universidade Federal Fluminense. Niterói. Rio de Janeiro, Rio de Janeiro, Brazil

**Keywords:** Healthcare Personnel, COVID-19, Pandemics, Workplace Violence, Working Conditions, Personal de Salud, COVID-19, Pandemias, Violencia en el Trabajo, Condiciones Laborales

## Abstract

**Objectives:**

to analyze how symbolic relationships established by healthcare professionals were expressed in their work during the pandemic.

**Methods:**

This qualitative study was conducted through an online survey. Participants were healthcare professionals working in the state of Rio de Janeiro, recruited between 07/20 and 09/30/2020. Data were processed using IRaMuTeQ software and discussed in light of Bourdieu’s concepts.

**Results:**

healthcare professionals experienced feelings such as fear, exhaustion, devaluation, stress, and burnout. They developed skills to confront SARS-CoV-2 but suffered from symbolic violence, often imperceptible yet real.

**Final Considerations:**

the impact of COVID-19 on the lives of healthcare professionals could have been mitigated with mechanisms to protect physical and mental health, in addition to guaranteeing labor rights and working conditions.

## RESUMEN

**Objetivos:**

analizar cómo se expresaron las relaciones simbólicas establecidas por los profesionales de salud en su ejercicio laboral durante la pandemia.

**Métodos:**

estudio cualitativo realizado mediante una encuesta en línea. Los participantes fueron profesionales de salud que trabajaban en el estado de Río de Janeiro, reclutados entre el 20/07 y el 30/09/2020. Los datos fueron procesados con el software IRaMuTeQ y discutidos a la luz de los conceptos de Bourdieu.

**Resultados:**

los profesionales de salud experimentaron sentimientos como miedo, agotamiento, desvalorización, estrés y cansancio extremo. Desarrollaron habilidades para enfrentar el SARS-CoV-2, pero sufrieron violencia simbólica, muchas veces imperceptible, pero real.

**Consideraciones Finales:**

el impacto de la COVID-19 en la vida de los profesionales de salud podría haberse mitigado con mecanismos de protección a la salud física y mental, además de la garantía de los derechos laborales y las condiciones de trabajo.

## INTRODUCTION

During the COVID-19 pandemic, the term “frontline” was widely used to refer to professionals in essential service areas (healthcare, public security, industry, commerce, etc.) who, due to the nature of their work at the time, faced greater risks of exposure to the SARS-CoV-2 virus and, consequently, higher risks of infection. Historically, this term strongly correlates with others such as “field hospitals”, “campaign hygienism”, and “medical and sanitary police”, which emerged in the early 20th century during the period of the Vaccine Revolt and the sanitization of ports in Brazil^(1)^. In the context of the armed forces, this term is considered a measure of space control and combat, marking friendly or enemy forces present in the battle zone during an armed conflict or war. When applied to the reality of fighting the COVID-19 pandemic, the “frontline” symbolizes the position occupied by healthcare professionals and all those who played essential roles in addressing the pandemic, engaging in strategic planning, information control for combating the SARS-CoV-2 virus, diagnosis, management, and care of sick patients^(2)^.

The pandemic became a calamity that directly impacted health, the living conditions of millions of people, the economy, and the organization of various countries. Over nearly three years of the pandemic (up to 05/01/2023), 388 million COVID-19 cases were reported worldwide, with 26 million in Brazil, accounting for 6.7% of all cases. During this period, 5.71 million COVID-19 deaths were recorded globally, with more than 630,000 in Brazil, representing 11% of the total^(3)^.

In Brazil, it was observed that the pandemic exacerbated vulnerability across different territories and populations^(3)^. The government’s difficulty in implementing national public policies capable of preventing crises or mitigating their consequences compromised the country’s development during the pandemic^(4)^. The lack of coordination by the federal government affected communication among authorities, hindering an immediate response to the health system and population demands.

With the persistence of the pandemic and national management issues, healthcare professionals faced a host of experiences, including illnesses inherent to healthcare work, constant exposure to suffering and end-of-life care, higher risks of exposure and infection, pandemic-related insecurities, and the emergence of new variants. Additionally, Brazil faced specific challenges such as delays in the approval, purchase, and distribution of vaccines, the absence of central coordination to address the pandemic, and political manipulation of the situation, resulting in misinformation about effective protection and control measures. This context created a scenario in which healthcare professionals were subjected to long working hours, high psychological stress, and physical fatigue^(5)^.

On May 5, 2023, the World Health Organization declared the end of the Public Health Emergency of International Concern regarding COVID-19^(6)^. However, there remains concern about the number of cases; as of August 13, 2023, there were 769,774,646 confirmed cases and 6,955,141 deaths from SARS-CoV-2 worldwide^(7)^. With the persistence of new cases and, consequently, the ongoing physical and mental burden on healthcare professionals due to the stressors generated by COVID-19, it is valid and justifiable to present the accounts of these frontline care professionals.

The limited publication of real data on the experiences of healthcare professionals during the pandemic hinders the creation of effective policies and adequate support, undermining the response to future crises.

## OBJECTIVES

To analyze how symbolic relationships established by frontline healthcare professionals during the COVID-19 pandemic were expressed in their work.

## METHODS

### Ethical Aspects

The study was conducted in accordance with national and international ethical guidelines and was approved by the Research Ethics Committee of the National Institute of Women’s, Children’s, and Adolescents’ Health Fernandes Figueira of FIOCRUZ (IFF/FIOCRUZ), whose decision is attached to this submission. Informed consent was obtained from all individuals involved in the study through online means.

### Study Type

This was a qualitative study conducted through an online, voluntary, and anonymous survey. Participants were healthcare professionals active during the pandemic in the state of Rio de Janeiro.

### Mode of Contact

The electronic form was initially distributed through instant messaging applications such as WhatsApp. Subsequently, the dissemination of the research expanded to social networks like Facebook and Instagram, and it was also promoted on the IFF/FIOCRUZ website.

### Study Setting

The study was carried out in the state of Rio de Janeiro, focusing on the period from July 20 to September 30, 2020. As of the study’s start date, the State Health Department of Rio de Janeiro reported 141,005 confirmed COVID-19 cases and 12,161 deaths due to the virus^(8)^.

### Data Source

There was no initial calculation for sample size. The researchers distributed the questionnaire link among their contact lists and social media followers, as well as on the institutional website of IFF/FIOCRUZ. After completing the questionnaire, participants were encouraged to share the link with their contacts and followers, creating a “snowball” effect^(9)^. The objective of this approach was to broaden the dissemination of the research and reach a wider audience.

### Inclusion/Exclusion Criteria

The inclusion criteria were healthcare professionals aged 18 years or older, working in the state of Rio de Janeiro during the pandemic, and who responded to the open-ended question in the survey. The exclusion criteria were defined as retired professionals and questionnaires with incomplete or irrelevant responses. The study sample comprised all participants who responded to the open-ended question in the survey: “We would like to leave this space open for you to write about how you are feeling during this pandemic or to contribute with suggestions on issues that were not addressed in this study”.

### Data Collection Instrument

An electronic form was created using Google Forms, containing 30 closed-ended questions and one open-ended question. For this article, only data from the open-ended question were used, which was made available to participants after they read and confirmed the online informed consent (TCLE). This article adhered to the EQUATOR guidelines for health research production, utilizing the instrument for e-surveys, the Checklist for Reporting Results of Internet E-Surveys (CHERRIES).

### Variables

The variables used in this study were: sex (male and female), age (stratified into 18-39 years, 40-59 years, and ≥60 years), and professional category (social worker, biologist/biomedical professional, nurse, pharmacist, physiotherapist, speech therapist, physician, nutritionist, psychologist, occupational therapist, dentist, nursing technician and/or assistant, radiology technician, laboratory technician, and others).

### Data Analysis

The IRaMuTeQ software (*Interface de R pour les Analyses Multidimensionnelles de Textes et de Questionnaires*) was used to process the data. This software enables textual analyses by organizing vocabulary distribution to facilitate data comprehension and visualization through correspondence analysis, similarity analysis, and word clouds^(10)^. The word cloud analysis generated by IRaMuTeQ grouped words and displayed them graphically based on their frequency. This lexical analysis provides an initial overview of the textual material’s content^(10)^.

### Linking IRaMuTeQ with Pierre Bourdieu’s Concepts

Pierre Bourdieu’s concepts were applied to elaborate and interpret the lexical analyses. According to Bourdieu, relationships established among individuals do not solely stem from shared objective relations or socioeconomic spaces but are rooted in symbolic exchanges that perpetuate recognition and proximity relationships^(11)^. Bourdieu describes symbolic violence as a situation of domination imposed through an order sustained by *habitus*, field, practice, and capitals, where social agents occupying dominant and subordinate positions within the field accept the existing order and practices as “natural”. Thus, symbolic systems fulfill their political function as instruments of imposition or legitimation of domination, contributing to ensuring one class’s dominance over another^(12)^.

## RESULTS

For the analysis of the relationships established on the frontline of the COVID-19 pandemic, accounts from 554 healthcare professionals who met the study’s inclusion criteria were considered. The majority of participants were female (85.38%), aged between 40 and 59 years (55.05%), and included physicians (39.53%), nurses (20.76%), and nursing technicians/assistants (12.09%).

**Figure 1:**
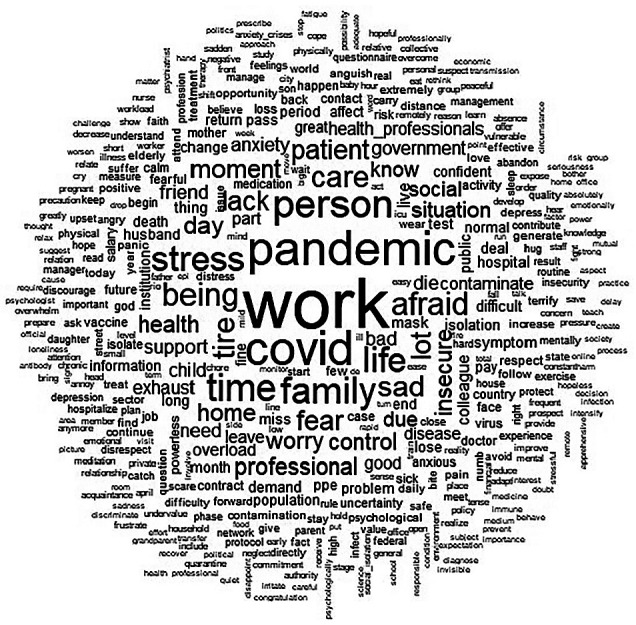
Word Cloud, Rio de Janeiro, Brazil, 2020 (N=554)

*Source: IRaMuTeQ Software*.

Considering the discourses of healthcare professionals examined using IRaMuTeQ, it was observed, through a word cloud (Figure 1), that the most frequently evoked words, with a frequency greater than 10, were: work, pandemic, COVID-19, fear, moment, person, family, stay, tired, see, life, sad, lack, patient, care, stress, and insecurity.

The prominence of the word “work” in this word cloud reflects the commitment of healthcare professionals during the pandemic in their professional duties. The pandemic demanded a level of dedication from healthcare professionals in caring for infected patients and combating the SARS-CoV-2 virus that far exceeded the protection, support, and guarantees of rights they received in return. The analysis of these professionals’ accounts highlighted the paradox between exposure and protection.

Healthcare professionals emphasized their fear. They also pointed out that many adverse outcomes related to COVID-19 could have been avoided or mitigated if the government had been more proactive and less negligent in implementing essential measures to combat the pandemic. Healthcare professionals feared becoming ill, infecting their family members or close contacts, and, above all, losing them (death due to COVID-19), in addition to experiencing feelings of insecurity.

*I feel insecure and afraid of contaminating myself and my family.* (Physician, female, 40-59 years old)

*Afraid of contracting the virus, developing a severe form of the disease, and dying, or worse, passing it on to my children, mother, spouse, or even acquaintances, and seeing them worsen and die.* (Nurse, female, 40-59 years old)

*Worried about the chaos the country is experiencing and the countless deaths that could have been avoided.* (Psychologist, female, 60≥ years old)

*I feel abandoned by the state, with no effective public policies to deal with the pandemic. A completely unprepared and criminal federal government is causing harm.* (Psychologist, stressed, female, up to 39 years old)

Being in the healthcare field required professionals to care for other lives, often at the expense of their own. Remaining in their field of work can almost be seen as a mission, a responsibility assumed throughout their careers. It is an enduring position of clinical self-sacrifice exercised by healthcare professionals^(13)^.

*I am worried about the possibility of getting sick, being hospitalized alone, and possibly dying. I have a chronic illness, but I chose not to step away at this time when the population needs healthcare services the most.* (Physician, male, 40-59 years old)

*The pandemic is known through this invisible, underappreciated, and rarely reported work. We also fear getting sick and bringing the virus home, losing team members who become ill, but we must stay strong.* (Nurse, female, 40-59 years old)

There were also concerns about labor rights, income loss, lack of payment, low wages, and unemployment.

*Lack of recognition for work overload. Lack of a bonus for being on the frontline. Suspension of vacations and days off during this difficult period of stress and loss of coworkers, and the suspension of labor rights such as tenure counting, premium leave, and loss of night shift differentials for professionals on leave due to chronic diseases or COVID-19.* (Nurse, female, 40-59 years old)

*Even though it is not my case, it angers me to see healthcare professionals working without a career plan, with low wages, and especially without job security.* (Physiotherapist, male, up to 39 years old)

*Extremely exhausted and underappreciated by our superiors. Unfortunately, there are no rights for us doctors, often not even the scheduled compensation, and this is frequent and discouraging, worsening our mental health already deteriorated by daily work.* (Physician, female, up to 39 years old)

*Due to the decline in pediatric care, I was let go and have been unemployed since May.* (Physician, male, 60≥ years old)

Participants’ statements highlighted vulnerability, lack of training, insufficient PPE, lack of transportation support for work, and institutional neglect.

*In my opinion, the issue of information and training about COVID at my workplace was the critical factor that led to the contamination of colleagues, as well as the late provision of PPE*. (Nutritionist, female, 40-59 years old)

*I feel extremely stressed due to difficulties in commuting to work, and I do not see my institution making an effort to help, with some exceptions.* (Nursing technician or assistant, female, 40-59 years old)

Changes in work routines and the lack of protocols caused stress.

*The greatest stress is the lack of a global protocol. Each physician prescribes as they see fit within a public institution, making it difficult to standardize due to the high turnover of these professionals.* (Pharmacist, female, up to 39 years old)

Professionals also experienced a lack of support for caring for their children due to isolation measures and family care responsibilities.

*We are in a situation where we often cannot provide ideal mental health care for our children. There is a dilemma involving the relationship between parents and grandparents. Either your children stay with the grandparents without you, or they stay with you without them. We cannot find anyone to care for our children.* (Physiotherapist, female, up to 39 years old)

*It was very difficult to go through this period alone, with two children, working a lot, with only the help of my nanny, far from my parents, siblings, and friends.* (Physician, female, 40-59 years old)

Healthcare professionals also expressed apprehension not only about their professional category but mainly about the disorganized government response, denialism by authorities, and the lack of a future perspective or direction in addressing the COVID-19 pandemic, leading to feelings of concern, dissatisfaction, and helplessness.

*Worried about the mismanagement the country is experiencing and the countless deaths that could have been avoided.* (Psychologist, female, 60≥ years old)

*I feel powerless about the situations generated by the pandemic, frustrated with government leaders and health policies.* (Nurse, female, 40-59 years old)

*No future perspective.* (Occupational therapist, female, 60≥ years old)

*Powerless and saddened by the ineffective and corrupt health policy at the federal, state, and municipal levels, denialism, and neglect of the population.* (Physician, female, 60≥ years old)

The image constructed before the pandemic by leaders, government officials, and public authorities in charge of the country’s management was significantly affected during the pandemic:

*To see so much negligence from authorities who remain inert in so many situations, failing to address those who truly need a different perspective and attention, acting with disregard, spreading misinformation, and engaging in corruption, ultimately leaving countless people to die without even a chance at treatment.* (Nursing technician or assistant, female, 40-59 years old)

*I am saddened to see our country without a competent Minister of Health, someone with a background in public health. We have no concrete and uniform measures. I am saddened by the loss of many classmates. I am doing my part to help, overcoming difficulties with caution.* (Physician, male, 60≥ years old)

These findings reflect the impact on healthcare professionals in various aspects of their lives, exacerbated by a lack of future prospects. This symbolizes a struggle, at that time, with no foreseeable end, which consequently led to discouragement, anguish, and depressive feelings.

## DISCUSSION

Analyses indicated that healthcare professionals working during the COVID-19 pandemic experienced fear, professional devaluation, stress, insecurity, concern, demotivation, helplessness, and physical and emotional exhaustion. The connections between these feelings and the symbolic relationships described align with Pierre Bourdieu’s concepts; thus, they will be used to structure the discussions in this article.

The results highlighted fear of becoming infected and transmitting the virus to their families as the predominant sentiment among healthcare professionals. This fear, combined with uncertainties regarding virus management and its global health repercussions, was especially pronounced at the start of the pandemic^(14)^.

Different healthcare professional categories, despite having varying levels of institutionalized cultural capital, reported fear in similar contexts both within their professional fields and their family lives. This type of institutionalized cultural capital refers to accumulated educational qualifications as well as incorporated cultural capital, which is acquired and internalized through long-term investments, becoming an integral part of the individual (agent)^(11)^.

The federal government, under the president’s leadership, displayed scientific denialism and authoritarianism, adopting an anti-science and militaristic rhetoric that resulted in symbolic violence against healthcare professionals^(4)^. This created a situation of domination imposed by the federal sphere, with the president (a social agent) occupying a dominant position in the field in which these professionals operated. With disdainful remarks about COVID-19 and disregard for scientific guidelines, the leadership contributed to high mortality rates in 2020^(15)^. The disqualification of scientific measures and the ridicule of healthcare professionals not only undermined their efforts but also reinforced a coercive dominance that directly impacted medical practice. The lack of recognition and the dismissal of healthcare professionals’ dedication constituted a form of symbolic violence, exacerbating the challenges faced in combating the pandemic.

The devaluation of healthcare professionals was tangible. A regrettable example of this disregard was the delay in approving the nursing wage floor. This category, one of the hardest-hit during the pandemic, remained for a long time without the right to fair wages. The first step toward institutionalizing the nursing wage floor occurred with the approval of Constitutional Amendment (EC in Portuguese) 124. Subsequently, on August 6, 2022, Law No. 14,434 was enacted, establishing the National Nursing Wage Floor for nurses, technicians, nursing assistants, and midwives, with a value of R$ 4,750 for nurses and scaled values for other categories^(16,17)^. However, the Supreme Federal Court (STFin Portuguese)) deemed the nursing wage floor’s approval unfeasible and suspended Law 14,434/22. Throughout this process, other laws and decrees were issued, and almost two years after negotiations began, on April 29, 2024, the matter remains unresolved, with the categories involved still awaiting the effective implementation of this wage^(18)^. Recognizing the worth of these professionals while denying them access to wage security is deeply frustrating.

Worldwide, nursing placed itself at the forefront of care during the pandemic, exposing the physical, mental, and familial health of its professionals, often resulting in a sense of abandonment^(19)^. In Brazil, it was no different. Aggravating the working conditions of Brazilian nurses even further, there was significant mobilization for the recognition of their role during the pandemic. However, this recognition did not materialize adequately, as even the vote on the wage floor was not authorized by the Senate.

It is necessary to consider that nursing, in addition to performing continuous work during the pandemic, both before and after it, has never received its due recognition-not due to a lack of resources to pay the wage floor but possibly because the profession is predominantly female, with lingering sociological traces of the devaluation of women and the endorsement of patriarchal norms^(20)^.

There were also reports of professionals who continued their work despite their own fears, comorbidities, and losses. This commitment to caring for life stems from the *habitus* acquired throughout their personal and professional journeys. Healthcare professionals, through their incorporated *habitus*, felt a responsibility to continue working in the field they occupied. Bourdieu defined *habitus* as acquired knowledge, an embodied, enduring, and transferable disposition resulting from a long process of learning and contact with various social structures^(21)^.

The COVID-19 pandemic represents one of the greatest global health challenges of the 21st century. There was a surge in demand for hospital beds, difficulties with human resources, and significant changes in work schedules, an increased pace, overtime hours, daily workloads, and the adoption of low wages and multi-job arrangements^(22)^. The changes resulting from the pandemic created insecurity, fatigue, stress, and anxiety among healthcare professionals, which may have directly impacted their performance within the social sphere. There was an expectation for these professionals to receive recognition through support and security, training and professional development, stability, better salaries, and improved working conditions; however, little was implemented^(23)^.

The lack of personal protective equipment (PPE), unified protocols, vaccines, support services, staff training, diagnostic materials, oxygen, and mechanical ventilators for critically ill patients, as well as the shortage of mediumand high-complexity hospital beds at the onset of the pandemic, contributed to the increase in COVID-19 cases^(24)^.

Financial instability, wage cuts, concerning levels of job insecurity, devaluation, and the loss of labor rights during the height of the pandemic were stressors mentioned in other studies^(25,26)^. Financial instability and losses were reported by frontline healthcare professionals who felt disadvantaged by temporary work contracts, delayed payments, and sudden layoffs^(27)^. Working conditions in Brazil have gradually been devalued, with a loss of social and labor rights, an increase in unemployment, underutilization of the workforce, and growing job precarity, especially following the 2017 Labor Reform (Law No. 13.467/2017)^(28)^.

In the past two decades, significant changes have been implemented in the Brazilian work environment, with clear indications of wage-based employment linking salaries to various forms of compensation, such as shifts, hourly work, short-term, and temporary contracts. These changes have resulted in job precarity, a lack of institutional attachment, multiple jobs, and job insecurity^(29)^.

Healthcare professionals have historically been responsible for “caring” and “saving lives”, roles that were heavily impacted by COVID-19-not due to a lack of desire or initiative on their part, but because of the unknowns surrounding how to manage this new virus and its effects on public health.

Despite having a largely extensive institutionalized cultural capital, this knowledge was not sufficient to immediately cure and prevent the deaths of patients with the disease. This situation was especially evident at the beginning of the pandemic, when there were neither vaccines nor effective treatments available to manage this previously unknown disease. These professionals experienced a misalignment between their desire to provide comprehensive care for COVID-19 patients and the limitations imposed by the national health system, which also affected their *habitus*. The act of caring and healing is an integral part of these professionals’ *habitus*, incorporated throughout their lives and careers.

Various internal resources were utilized by these professionals in their efforts to combat the virus and care for the sick. These resources were primarily derived from institutionalized cultural capital, referring to accumulated educational qualifications, and incorporated cultural capital, which is acquired and internalized through long-term investments, becoming a part of the individual (agent)^(11)^. The cultural capital that healthcare professionals possessed prior to the pandemic was revived and applied to navigate the challenges of the pandemic within their field. They acted with altruism, a characteristic inherent to the profession.

The issue of prolonged exposure to stressors among healthcare professionals also emerged. Occupational stress was evident in their field, with demands that exceeded their coping capacity, resulting in negative impacts on their health and well-being. The pandemic exposed a significant vulnerability among healthcare professionals, along with continuous exposure to external factors such as biological, economic, and structural risks, as well as individual factors^(30,31)^.

The group of professionals who worked on the frontline of care comprised social agents who, for the most part, operated in hospital settings. This environment is dynamic and unstable, where knowledge is gained through contact with various social structures, allowing the agent to adapt and evolve naturally, characterizing their professional *habitus*. The professional *habitus* of this group was formed through repeated exposure to the specific conditions of the hospital environment, instilling in them a set of durable and transferable dispositions internalized through the need to remain and situate themselves within this social universe^(11)^.

The hospital environment during the COVID-19 pandemic was organized into a field characterized by agents possessing a shared *habitus*. Within this occupied field, whether in a public or private setting, changes in routines and impositions of power were identified. The power imposed by federal authorities led to dominance within the field in both settings. Drawing on the *habitus* and capital each healthcare professional had incorporated throughout their social and professional journeys, they were able to remain active during the COVID-19 pandemic. This environment (social space) in which agents positioned themselves was reorganized to meet the high demand imposed by the pandemic. Transformations were carried out to offer the best possible promotion, protection, recovery, and rehabilitation of health to the population, but they had repercussions on multiple dimensions^(32)^.

The agents within the field developed strategies that adapted to the pandemic’s situation. Even without full awareness, the agents (healthcare professionals) acquired a practical sense through continued exposure to similar situations, thereby internalizing different ways of acting^(11)^. The structures acquired within the field became forms of capital. Those who underwent targeted training and capacity-building for COVID-19 care experienced changes in their actions, practices, and even their positions within the hospital field due to newly incorporated capital. It was noted that the evolution of a professional’s position depends on their capital and the strategies they implement. Healthcare professionals collectively sought recognition and better positions within the hospital field during the pandemic without highlighting one category over another.

Another challenge faced by healthcare professionals was the issue of childcare, especially because schools remained closed for a long period in Brazil. Many elderly individuals who were part of healthcare professionals’ support networks needed to avoid contact. As a result, many professionals had to work while simultaneously caring for their children, making logistics more difficult and complicated^(33)^.

Healthcare professionals sought an unequivocal guarantee that their organizations would support both them and their families. These professionals desired visible leadership during this turbulent period and recognition for their work^(34)^. However, the data indicate that this support was not perceived by healthcare professionals, regardless of their professional category.

Reports of vacation suspensions were verified. It is worth noting that, under Provisional Measure No. 927/2020, dated March 22, 2020, in Article 7, the suspension of vacations or unpaid leave for healthcare professionals or those performing essential functions during the public calamity period served the public interest, which supersedes individual interests.

Participants also highlighted feelings of powerlessness, the need for public policies, and government actions. The COVID-19 pandemic in Brazil became a critical event of multiple proportions, with repercussions exacerbated by the disastrous combination of a president characterized as genocidal and a government driven by economic interests^(35)^. The symbolic power exercised by leaders responsible for pandemic-related decisions was determined by the words spoken by the health ministers and the president of the republic.

Bourdieu (2004) asserts that symbolic hierarchy reinforces social hierarchy through the division between dominant and dominated groups based on the volume and types of capital accumulated. The capital of these professionals did not hold weight in determining their social positions, as it was not the capital itself but the symbolic hierarchy to which they were subjected that dictated their social standing^(36)^.

In the analysis of the study data, no reports of power and domination relationships were observed among professional categories, which could have occurred given that these categories possess differentiated cultural and even specific forms of capital. This might be understood as a need for mutual support within the context of the pandemic. However, the power and symbolic hierarchy exerted by the State were evident, generating feelings of powerlessness and anxiety among frontline healthcare workers. The text segments expressed by professionals highlighted feelings of concern and dissatisfaction due to the lack of support from management. The main concerns of this group included insufficient institutional support and the behavior of some government officials and state employees who underestimated the severity of the pandemic, contributing to the spread of COVID-19 and leading to a sense of insecurity, anxiety, and a lack of hope for a better future^(25,37)^.

There was a coordinated response to the Coronavirus disease with the activation, on 01/22/2020, of the Public Health Emergency Operations Center (COE-COVID19), which provided case definitions, guidance for reporting, laboratory investigations, isolation and transportation measures, as well as patient care in primary and specialized settings, and hygiene measures^(37)^. Among its objectives, the SUS (Unified Health System) aimed to establish swift mechanisms for case registration, acquisition, distribution, assessment, and monitoring of the quality of products, equipment, and supplies related to the pandemic response, as well as implementing guidelines to minimize the impact and spread of the pandemic^(38)^.

In practice, however, there was no central coordination, leading to a sense of disorder for those on the frontline of care. Feelings of powerlessness, frustration, and lack of reliable information were not confined to a specific sector, nor were they limited to public or private settings, a single professional category, or only those with less accumulated capital.

The pandemic led to physical exhaustion among healthcare professionals. Various aspects were discussed, with terms like “tired”, “exhausted”, “worn out”, and “without hope” standing out, even in the word cloud. By virtue of their *habitus*, healthcare professionals remained active in their field and drew on their incorporated cultural capital. However, prolonged exposure to SARS-CoV-2 and the continuous impact of COVID-19 affected not only their emotional well-being but also their physical health.

Examining the highlighted central points reveals convergence in a form of symbolic violence directed by the federal entity (dominating) toward healthcare professionals (dominated) who were on the frontline of care during the COVID-19 pandemic. Different healthcare categories, regardless of their *habitus* and accumulated capital, shared a recognition of the need for a resolute change within the field during the pandemic. Yet, due to their subordinate position, they were unable to transform this reality.

### Study limitations

There was a higher response rate to the questionnaire from physicians and nurses due to the researchers’ professional networks, which primarily consist of individuals from these categories. The same holds true regarding the predominance of female respondents, as the researchers involved in this study work in women’s and children’s health, where female representation is significant. This situation limits the extrapolation of the results to other profiles but, on the other hand, allows for a more precise analysis of this group, which is highly representative among healthcare professionals.

### Contributions to the Nursing Field

This research significantly contributes to nursing by highlighting the impacts of COVID-19 on the physical and mental health of healthcare professionals in Rio de Janeiro. It identifies feelings such as fear and exhaustion, emphasizing the need for comprehensive care. The analysis of symbolic relationships sheds light on often imperceptible forms of violence. The conclusion underscores the importance of measures to protect health and labor rights and promote the well-being of healthcare professionals.

## FINAL CONSIDERATIONS

By gathering and interpreting the statements of healthcare professionals, it was possible to identify different forms of violence experienced by them in their work environment and surrounding context. Using Bourdieu’s theoretical framework, it was observed from these professionals’ accounts that within a dynamic field, *habitus*, cultural capital, and strategies adopted by agents were decisive for their persistence and survival amid symbolic violence experienced during the pandemic. Symbolic violence was exercised through symbolic power imposed silently on healthcare professionals (dominated), permeating their field of work. As support, working conditions, and material resources were denied to confront the pandemic, symbolic violence was exerted-often imperceptible but very real-creating limitations within the dominated class. This form of violence, insidious in nature, is primarily exercised through symbolic channels of communication and knowledge, with actions that subject the dominated. These analyses revealed that various healthcare professionals, while performing their work functions, faced hardships in the form of often undeclared symbolic violence.
