# Supplementary material for: Symbolic violence experienced by frontline healthcare professionals during the COVID-19 pandemic
Source: Rev Bras Enferm. 2025 Apr 28;78(2):e20240237. doi: 10.1590/0034-7167-2024-0237 (PMC12037186; doi:10.1590/0034-7167-2024-0237)
Supplement: Translation

# Translation: Violência simbólica vivida pelos profissionais de saúde da linha de frente da pandemia da COVID-19

**DOI:** 10.1590/0034-7167-2024-0237pt

**Authors:** Karla Gonçalves Camacho, Saint Clair dos Santos Gomes, Maria de Fátima Junqueira-Marinho, Adriana Teixeira Reis, Dimitri Marques Abramov, Daniella Campelo Batalha Cox Moore

**Affiliations:** I Instituto Nacional de Saúde da Mulher, da Criança e do Adolescente Fernandes Figueira, Fundação Oswaldo Cruz. Rio de Janeiro, Rio de Janeiro, Brasil; II Universidade do Estado do Rio de Janeiro. Rio de Janeiro, Rio de Janeiro, Brasil; III Universidade Federal Fluminense. Niterói. Rio de Janeiro, Rio de Janeiro, Brasil

**Keywords:** Pessoal de Saúde, COVID-19, Pandemias, Violência no Trabalho, Condições de Trabalho

## Abstract

**Objetivos:**

analisar de que forma se expressaram relações simbólicas estabelecidas pelos profissionais de saúde no exercício laboral durante a pandemia.

**Métodos:**

estudo qualitativo, realizado por meio de um inquérito online. Os participantes foram profissionais de saúde atuantes no estado do Rio de Janeiro, recrutados entre 20/07-30/09/2020. Os dados foram processados pelo software IRaMuTeQ e discutidos à luz dos conceitos de Bourdieu.

**Resultados:**

os profissionais de saúde vivenciaram sentimentos como medo, cansaço, desvalorização, estresse e esgotamento. Desenvolveram habilidades para enfrentar o SARS-CoV-2, mas sofreram violência simbólica, muitas vezes imperceptível, porém real.

**Considerações Finais:**

o impacto da COVID-19 na vida dos profissionais de saúde poderia ter sido amenizado com mecanismos de proteção à saúde física e mental, além da garantia dos direitos trabalhistas e condições de trabalho.

## INTRODUÇÃO

Durante a pandemia da COVID-19, o termo “linha de frente” foi amplamente usado para se referir aos profissionais das áreas de serviços considerados essenciais (saúde, segurança pública, indústria, comércio etc.) que, devido à própria natureza das atividades que desempenhavam à época, estavam em maior risco de exposição ao vírus SARS-CoV-2 e, consequentemente, em maior risco de infecção. Historicamente, esta expressão dialoga fortemente com outros termos, como “hospital de campanha”, “higienismo campanhista” e “polícia médica e sanitária”, que surgiram no início do século XX, durante o período da Revolta da Vacina e da higienização dos portos no Brasil^(1)^. No contexto das forças armadas, esse termo é considerado como uma medida de controle de espaço e de luta, demarcando forças amigas ou inimigas presentes na zona de batalha durante um conflito armado ou guerra. Transpondo para a realidade do combate à pandemia da COVID-19, a “linha de frente” simboliza a posição ocupada pelos profissionais de saúde e por todos que desempenharam funções imprescindíveis no enfrentamento à pandemia, com ações de planejamento estratégico, controle de informações no combate ao vírus SARS-CoV-2, diagnóstico, gerenciamento e cuidado de pacientes doentes^(2)^.

A pandemia tornou-se uma calamidade que afetou diretamente a saúde, as condições de vida de milhões de pessoas, a economia e a organização de diferentes países. Nos quase três anos de permanência da pandemia (até 01/05/2023), foram registrados 388 milhões de casos de COVID-19 no mundo e 26 milhões no Brasil, correspondendo a 6,7% de todos os casos. Nesse período, registraram-se 5,71 milhões de óbitos por COVID-19 no mundo e mais de 630 mil no Brasil, equivalendo a 11% do total^(3)^.

No Brasil, observou-se que a pandemia ampliou a vulnerabilidade em diferentes territórios e populações^(3)^. A dificuldade do governo em programar políticas públicas nacionais capazes de prevenir crises ou mitigar suas consequências comprometeu o desenvolvimento do país durante a pandemia^(4)^. A descoordenação do governo federal afetou a comunicação entre os poderes, impedindo uma resposta imediata às demandas do sistema de saúde e da população.

Com a persistência da pandemia e dos problemas gerenciais nacionais, os profissionais de saúde enfrentaram uma soma de experiências, como o adoecimento inerente ao trabalho em saúde, o contato constante com o sofrimento e a finitude da vida, maior risco de exposição e contaminação, inseguranças próprias da pandemia e o surgimento de variantes. O Brasil apresentou, ainda, situações adicionais, como a lentidão na aprovação, compra e distribuição das vacinas, a falta de uma articulação central para o enfrentamento da pandemia e o uso político da situação, gerando desinformação sobre medidas eficazes de proteção e controle. Todo esse contexto criou um cenário em que os profissionais de saúde foram submetidos a longas horas de trabalho, altas cargas de sofrimento psicológico e desgaste físico^(5)^.

A Organização Mundial da Saúde declarou, em 5 de maio de 2023, o fim da Emergência de Saúde Pública de Importância Internacional referente à COVID-19^(6)^. Contudo, ainda há preocupação com o número de casos dessa doença; até 13 de agosto de 2023, havia 769.774.646 casos confirmados e 6.955.141 mortes pelo SARS-CoV-2^(7)^. Com a persistência de novos casos e, consequentemente, a contínua sobrecarga física e mental dos profissionais de saúde devido aos fatores estressantes gerados pela COVID-19, torna-se válido e justificável apresentar os relatos desses profissionais da linha de frente do cuidado.

A reduzida publicação de dados reais sobre as experiências dos profissionais de saúde durante a pandemia dificulta a criação de políticas eficazes e o suporte adequado, prejudicando a resposta a futuras crises.

## OBJETIVOS

Analisar de que forma se expressaram as relações simbólicas estabelecidas pelos profissionais de saúde da linha de frente na pandemia da COVID-19 em seu exercício laboral.

## MÉTODOS

### Aspectos Éticos

O estudo foi conduzido de acordo com as diretrizes éticas nacionais e internacionais e aprovado pelo Comitê de Ética em Pesquisa do Instituto Nacional de Saúde da Mulher, da Criança e do Adolescente Fernandes Figueira da FIOCRUZ (IFF/FIOCRUZ), cujo parecer está anexado à presente submissão. O Consentimento Livre e Esclarecido foi obtido de todos os indivíduos envolvidos no estudo por meio online.

### Tipo de Estudo

Trata-se de um estudo qualitativo, realizado por meio de um inquérito *online*, voluntário e anônimo. Os participantes foram profissionais de saúde atuantes na pandemia, no estado do Rio de Janeiro.

### Modo de Contato

Optou-se por distribuir o formulário eletrônico inicialmente por aplicativos de mensagens instantâneas como *WhatsApp*. Em seguida, expandiu-se a divulgação da pesquisa em redes sociais como *Facebook* e *Instagram*, além de ser divulgado no site do IFF/FIOCRUZ.

### Cenário do Estudo

O cenário do estudo foi o estado do Rio de Janeiro, com um recorte da pandemia no período de 20 de julho a 30 de setembro de 2020. Na data de início do estudo, a Secretaria de Estado de Saúde do Rio de Janeiro registrava 141.005 casos confirmados e 12.161 óbitos por coronavírus (COVID-19)^(8)^.

### Fonte de Dados

Não houve cálculo inicial do tamanho da amostra. Os pesquisadores distribuíram o link com o questionário entre suas listas de contatos e seguidores nas redes sociais, bem como no site institucional do IFF/FIOCRUZ. Depois que os participantes completaram o questionário, foram encorajados a compartilhar o link com seus próprios contatos e seguidores, criando um efeito de “bola de neve”^(9)^. O objetivo dessa abordagem foi ampliar a divulgação da pesquisa e atingir um público mais amplo.

### Critérios de Inclusão/Exclusão

Os critérios de inclusão foram: profissionais de saúde, maiores de 18 anos, atuantes no estado do Rio de Janeiro no período da pandemia e que responderam à questão aberta do inquérito. Os critérios de exclusão definidos foram: aposentados e questionários com respostas contendo dados incompletos ou irrelevantes. A amostra do estudo foi composta por todos os participantes que responderam à questão aberta do inquérito: “Gostaríamos de deixar este espaço aberto para você escrever como se sente neste momento de pandemia ou contribuir com sugestões de questões que não foram contempladas neste estudo”.

### Instrumento de Coleta de Dados

Foi elaborado um formulário eletrônico no *Google Forms*, contendo 30 questões fechadas e uma questão aberta. Para este artigo, foram utilizados apenas os dados da questão aberta, cujo preenchimento era disponibilizado aos participantes após a leitura e confirmação do TCLE *online*. Este artigo respeitou as diretrizes para produção de pesquisa em saúde da EQUATOR, utilizando o instrumento para pesquisas do tipo *e-surveys, Checklist for ReportingResultsof Internet E-Surveys* (CHERRIES).

### Variáveis

As variáveis utilizadas neste estudo foram: sexo (masculino e feminino), idade (estratificada em 18-39 anos, 40-59 anos e ≥ 60 anos) e categoria profissional (assistente social, biólogo/biomédico, enfermeiro, farmacêutico, fisioterapeuta, fonoaudiólogo, médico, nutricionista, psicólogo, terapeuta ocupacional, odontólogo, técnico e/ou auxiliar de enfermagem, técnico em radiologia e em laboratório, outros).

### Análise dos Dados

Foi utilizado o software IRaMuTeQ (*Interface de R pourlesAnalysesMultidimensionnelles de Textes et de Questionnaires*) para processar os dados. Este software permite executar análises textuais, sendo capaz de organizar a distribuição do vocabulário de forma a facilitar a compreensão e visualização dos dados por meio de análises de correspondências, de similitude e nuvem de palavras^(10)^. A análise de nuvem de palavras gerada pelo IRaMuTeQ agrupou palavras e organizou-as graficamente em função de sua frequência. Trata-se de uma análise lexical que fornece uma ideia inicial do conteúdo do material textual^(10)^.

### Articulação do IRaMuTeQ com os Conceitos de Pierre Bourdieu

Os conceitos de Pierre Bourdieu foram aplicados para discorrer e interpretar as análises lexicais. Para Bourdieu, as relações estabelecidas entre os sujeitos não advêm unicamente do simples compartilhamento de relações objetivas ou de espaços socioeconômicos, mas ancoram-se em trocas simbólicas que permitem a perpetuação do reconhecimento e das relações de proximidade^(11)^. Bourdieu descreve a violência simbólica como uma situação de dominação imposta por meio de um ordenamento sustentado pelo *habitus*, campo, prática e capitais, onde os agentes sociais que ocupam posições de dominantes e dominados no campo aceitam a ordem e as práticas existentes como “naturais”. Dessa forma, os sistemas simbólicos cumprem sua função política de instrumentos de imposição ou de legitimação da dominação, contribuindo para assegurar a dominação de uma classe sobre outra^(12)^.

## RESULTADOS

Para a análise das relações estabelecidas na linha de frente da pandemia da COVID-19, foram considerados relatos de 554 profissionais de saúde que atenderam aos critérios de inclusão do estudo. A maioria dos participantes era do sexo feminino (85,38%), com idade entre 40 e 59 anos (55,05%), sendo médicos (39,53%), enfermeiros (20,76%) e técnicos/auxiliares de enfermagem (12,09%).

Considerando os discursos dos profissionais de saúde, examinados pelo IRaMuTeQ, verificou-se, por meio da nuvem de palavras (Figura 1), que as palavras mais evocadas, com frequência maior que 10, foram: trabalho, pandemia, COVID-19, medo, momento, pessoa, família, ficar, cansado, ver, vida, triste, falta, paciente, cuidado, estresse e inseguro.

**Figura 1:** Nuvem de palavras, Rio de Janeiro, Brasil, 2020 (N=554)

*Fonte: Software IRaMuTeQ.*

O destaque da palavra “trabalho” nesta nuvem reflete o envolvimento dos profissionais de saúde durante a pandemia em seu exercício laboral. A pandemia exigiu dos profissionais de saúde uma dedicação à assistência dos pacientes infectados e ao combate ao vírus SARS-CoV-2 em uma proporção superior ao retorno ofertado em termos de proteção, suporte e garantia de direitos. A análise dos registros desses profissionais trouxe à tona o paradoxo entre exposição e proteção.

Os profissionais de saúde enalteceram o medo. Destacaram também que muitos desfechos ruins da COVID-19 poderiam ter sido evitados ou amenizados se o governo tivesse sido mais atuante e menos omisso em medidas fundamentais de combate à pandemia. Os profissionais de saúde tinham medo de adoecer, de contaminar seus familiares ou pessoas próximas, e, principalmente, de perdê-las (morte por COVID-19), além de sentirem insegurança.

*Me sinto insegura, com medo de contaminar a mim e à minha família* (Médica, sexo feminino, entre 40-59 anos)

*Com medo de contrair e chegar até a forma mais grave e morrer ou pior passar para meus filhos, mãe, ou esposo, ou até mesmo conhecidos e eles agravarem e morrerem.* (Enfermeira, sexo feminino, entre 40-59 anos)

*Preocupada com o desgoverno que o país está passando e as inúmeras mortes que poderiam ter sido evitadas* (Psicóloga, sexo feminino, 60≥ anos).

*Me sinto abandonada pelo estado sem políticas públicas efetivas para lidar com a pandemia um governo federal totalmente despreparado e criminoso está sendo adoecedor.* (Psicólogo,com estresse, sexo feminino, até 39 anos)

O fato de ser da área da saúde exigiu que o profissional cuidasse de outras vidas, muitas vezes em detrimento de sua própria vida. A permanência no campo de atuação pode ser compreendida quase como uma missão, uma responsabilidade assumida ao longo de sua trajetória. Trata-se de uma posição atemporal de auto-sacrifício clínico exercido pelos profissionais de saúde^(13)^.

*Estou preocupado com a possibilidade de adoecer e ser internado solitariamente e de vir a morrer, tenho doença crônica, mas optei por não me ausentar neste momento que a população mais precisa de atendimento na área da saúde.* (Médico, sexo masculino, entre 40-59 anos)

*A pandemia se conhece através desse trabalho invisível pouco valorizado e noticiado. Também temos medo de adoecer e levar o vírus para casa, perder pessoas da equipe que adoecem, mas precisamos permanecer firmes.* (Enfermeiro, sexo feminino, entre 40-59 anos)

Houve também preocupações com os direitos trabalhistas, perda de renda, falta de pagamento, baixa remuneração e desemprego.

*Ausência de reconhecimento pela sobrecarga de trabalho. Ausência de uma gratificação por estar na linha de frente. Suspensão de férias e folgas neste momento difícil de estresse e perda dos colegas de trabalho, suspensão de direitos trabalhistas como contagem de triênios, licença prêmio, perda de adicional noturno para os profissionais com afastamento por doenças crônicas ou COVID-19.* (Enfermeira, sexo feminino, entre 40-59 anos)

*Mesmo não sendo o meu caso, me revolta ver profissionais de saúde trabalhando sem plano de carreira, baixa remuneração e principalmente sem vínculo empregatício.* (Fisioterapeuta, sexo masculino, até 39 anos)

*Extremamente exausta e pouco valorizada por nossos superiores, infelizmente não há qualquer direito para nós médicos, frequentemente nem mesmo a remuneração programada, isso é frequente e desanimador, piora a nossa saúde mental já deteriorada pelo dia a dia de trabalho.* (Médico, sexo feminino, até 39 anos)

*Devido à baixa dos atendimentos pediátricos fui dispensado, estou desempregado desde maio.* (Médico, sexo masculino, 60≥ anos)

Falas dos participantes destacaram vulnerabilidade, falta de treinamento, de EPI, de apoio no transporte para o trabalho e desamparo das instituições.

*No meu entender a questão das informações e treinamento sobre o COVID, onde trabalho, foi o ponto crucial que levou a contaminação de colegas e também a questão do fornecimento tardio dos EPI.* (Nutricionista, sexo feminino, entre 40-59 anos)

*Me sinto extremamente estressada devido a dificuldades em locomoção para o trabalho e não vejo a instituição que trabalho se esforçando para ajuda com algumas exceções.* (Técnico ou auxiliar de enfermagem, sexo feminino, entre 40-59 anos)

As mudanças nas rotinas de trabalho e a falta de protocolos gerou estresse.

*O maior estresse é a falta de protocolo mundial, cada médico prescreve como acha melhor dentro de uma instituição pública, fica difícil padronizar por causa da alta rotatividade desses profissionais.* (Farmacêutico, sexo feminino, até 39 anos)

Os profissionais também vivenciaram a falta de apoio para o cuidado com os filhos, devido às medidas de isolamento e cuidado com os familiares.

*Estamos em um momento que muitas vezes não conseguimos proporcionar uma saúde mental ideal para nossos filhos, um dilema envolve o convívio entre pais e avós. Ou seus filhos ficam com os avós sem você, ou fica com você e sem eles. Não encontramos quem cuide dos nossos filhos.* (Fisioterapeuta, sexo feminino, até 39 anos)

*Foi muito difícil passar por esse período sozinha, com dois filhos, trabalho muito, com ajuda apenas da minha babá, longe dos meus pais, irmãos e amigos.* (Médica, sexo feminino, entre 40-59 anos)

Ressaltaram ainda, a apreensão dos profissionais de saúde não só com sua categoria profissional, mas principalmente com o desgoverno, negacionismo por parte do governo e falta de perspectiva de futuro, de um direcionamento diante da pandemia da COVID-19, gerando sentimentos de preocupação, insatisfação e impotência.

*Preocupada com o desgoverno que o país está passando e as inúmeras mortes que poderiam ter sido evitadas.* (Psicóloga, sexo feminino, 60≥ anos)

*Me sinto impotente com as situações geradas pela pandemia, frustrada com os governantes e as políticas de saúde.* (Enfermeiro, sexo feminino, entre 40-59 anos)

*Sem perspectiva futura.* (Terapeuta ocupacional, sexo feminino, 60≥ anos)

*Impotente e triste pela política de saúde ineficaz e corrupta a nível federal, nível estadual e nível municipal, negacionismo, negligência com a população.* (Médica, sexo feminino, 60≥ anos)

A imagem construída, antes da pandemia, pelas lideranças, governantes e autoridades responsáveis pela gestão pública do país foi afetada na pandemia:

*Ver tanto descaso de autoridades que se demonstram inerte a tantas situações e não olham para quem de fato necessita de um olhar diferenciado e atenção, agindo com descaso com tantas desinformações, sem contar com tanta corrupção, deixando que assim tantas pessoas fossem a óbitos sem a menor chance de tratamento.* (Técnico ou auxiliar de enfermagem, sexo feminino, entre 40-59 anos)

*Estou triste em ver nosso país sem um ministro da saúde competente, alguém com formação em saúde pública, não temos medidas concretas e uniformes estou triste por ter perdido vários colegas de turma faço minha parte tentando ajudar superando as dificuldades e com cautela.* (Médico, sexo masculino,60≥ anos)

Tais achados denotam o acometimento do profissional de saúde em várias esferas que o constituem, agravado pela falta de perspectiva futura. Isso simboliza uma luta, naquele momento, sem previsão de término, o que, consequentemente, gerava desestímulo, angústia e sentimentos depressivos.

## DISCUSSÃO

As análises indicaram que os profissionais de saúde atuantes na pandemia da COVID-19 vivenciaram medo, desvalorização profissional, estresse, insegurança, preocupação, desmotivação, impotência, esgotamento físico e emocional. As articulações entre esses sentimentos e as relações consideradas simbólicas se aproximam dos conceitos de Pierre Bourdieu; por isso, eles serão utilizados para encadear as discussões deste artigo.

Os resultados destacaram o medo de se infectar e transmitir o vírus à família como o sentimento predominante entre os profissionais de saúde. O medo associado ao vírus, às incertezas quanto ao seu manejo e às repercussões na saúde mundial foi enfatizado, especialmente no início da pandemia^(14)^.

As diferentes categorias profissionais da área da saúde, apesar de possuírem capital cultural institucionalizado diferenciado, relataram medo em situações similares dentro do campo de atuação e no contexto familiar. Esse tipo de capital, cultural institucionalizado, remete aos títulos escolares acumulados e também ao capital cultural incorporado, adquirido e internalizado por meio de investimentos de longa duração, que passaram a ser parte integrante da pessoa (agente)^(11)^.

O governo federal, sob a liderança do presidente, demonstrou negacionismo científico e autoritarismo, adotando um discurso anti-ciência e militarista que resultou em violência simbólica contra os profissionais de saúde^(4)^, uma vez que houve uma situação de dominação imposta pela esfera federal, com o presidente (agente social) ocupando uma posição de dominante no campo de atuação desses profissionais. Com declarações desdenhosas sobre a COVID-19 e desprezo pelas orientações científicas, a liderança contribuiu para altas taxas de mortalidade em 2020^(15)^. A desqualificação das medidas científicas e a ridicularização dos profissionais não apenas desconsideraram seus esforços, mas também consolidaram um domínio impositivo que afetou diretamente a prática médica. A ausência de reconhecimento e o menosprezo pela dedicação dos profissionais de saúde configuraram uma forma de violência simbólica, exacerbando as dificuldades enfrentadas no combate à pandemia.

A desvalorização dos profissionais de saúde foi real. Um exemplo triste desse desprestígio foi a demora na aprovação do piso salarial da enfermagem. Essa categoria, uma das mais atingidas na pandemia, permaneceu, por muito tempo, sem direito a um salário justo. O primeiro passo para a institucionalização do piso da enfermagem ocorreu com a aprovação da Emenda Constitucional (EC) 124. Logo em seguida, em 6 de agosto de 2022, foi instituída a Lei n.º 14.434, que estabelecia o Piso Nacional da Enfermagem para enfermeiras(os), técnicas(os), auxiliares de enfermagem e parteiras, no valor de R$ 4.750 para enfermeiros e valores escalonados para outras categorias^(16,17)^.

No entanto, o ministro do Supremo Tribunal Federal (STF) considerou inexequível a aprovação do piso salarial da enfermagem e suspendeu a Lei 14.434/22. Nesse percurso, outras leis e decretos foram publicados, e quase dois anos após o início das negociações, em 29 de abril de 2024, ainda há movimentações nessa questão; as categorias envolvidas aguardam a implementação efetiva desse salário^(18)^. Considerar justa a valorização desses profissionais, mas não permitir acesso a uma segurança salarial, é frustrante.

Em todo o mundo, a enfermagem expôs sua mão de obra ao assumir a linha de frente do cuidado na pandemia. A exposição da saúde física, mental e familiar desses profissionais muitas vezes resultou em um sentimento de abandono^(19)^. No Brasil, não foi diferente. Agravando ainda mais as condições de trabalho da enfermagem brasileira, houve uma grande mobilização pelo reconhecimento do papel desses profissionais durante a pandemia. Entretanto, esse reconhecimento não se concretizou adequadamente, pois nem a votação do piso salarial foi autorizada pelo Senado.

É necessário ponderar que a enfermagem, além de exercer um trabalho ininterrupto durante a pandemia, antes e depois dela, nunca recebeu o devido valor, não por falta de recursos para pagar o piso salarial, mas possivelmente pelo fato de a categoria ser eminentemente feminina, ainda havendo resquícios sociológicos de desvalorização da mulher e o endosso do patriarcado^(20)^.

Houve também registro de profissionais que permaneceram exercendo seu ofício, mesmo em detrimento de seus próprios medos, comorbidades e perdas. Esse comprometimento com o cuidado da vida é oriundo do *habitus* adquirido ao longo de sua trajetória de vida e profissional. O profissional de saúde, por seu *habitus* incorporado, sentia-se responsável por continuar atuando no campo que ocupava. Bourdieu definia *habitus* como um conhecimento adquirido, uma disposição incorporada, duradoura e transferível, resultante de um longo processo de aprendizado e produto do contato com diversas estruturas sociais^(21)^.

A pandemia da COVID-19 configura um dos maiores desafios sanitários em escala mundial no século XXI. Observou-se uma elevação da demanda por leitos, dificuldades com recursos humanos e profundas alterações quanto à jornada de trabalho, aumento do ritmo, das horas extras, da carga de trabalho diária e adoção de salários baixos e multiemprego^(22)^. As mudanças decorrentes da pandemia geraram insegurança, cansaço, estresse e angústia nos profissionais de saúde, o que pode ter impactado diretamente sua atuação dentro do espaço social. Esperava-se a valorização desses profissionais com suporte e segurança, treinamento e capacitação, além de estabilidade, melhores salários e condições de trabalho; contudo, pouco foi colocado em prática^(23)^.

A falta de equipamentos de proteção individual (EPI), de protocolos unificados, de vacinas, de serviços de apoio, de capacitação de pessoal, de materiais para diagnósticos, de oxigênio e de ventiladores mecânicos para pacientes críticos, bem como a carência de leitos hospitalares de média e alta complexidade no início da pandemia, contribuíram para o aumento no número de casos de COVID-19^(24)^.

A instabilidade financeira, os cortes salariais, os níveis preocupantes de insegurança no trabalho, a desvalorização e a perda de direitos trabalhistas no auge da pandemia foram fatores de estresse mencionados em outros estudos^(25,26)^. A instabilidade e as perdas financeiras foram relatadas por profissionais de saúde da linha de frente que se sentiram prejudicados por contratos de trabalho temporários, com atrasos nos pagamentos e demissões repentinas^(27)^. As condições de trabalho no cenário brasileiro vêm sendo, aos poucos, desvalorizadas, com perda de direitos sociais e trabalhistas, aumento do desemprego, subutilização da força de trabalho e crescente precarização das profissões, especialmente após a Reforma Trabalhista de 2017 (Lei nº 13.467/2017)^(28)^.

Nas últimas duas décadas, foram implementadas, no Brasil, mudanças importantes no ambiente de trabalho, com sinais claros de assalariamento, e com a vinculação do salário a diversas ofertas: por plantão, por hora trabalhada, contratos curtos e temporários, o que gera precarização, ausência de vinculação institucional, multiemprego e insegurança no ambiente de trabalho^(29)^.

Os profissionais de saúde historicamente são responsáveis pela função de “cuidar” e “salvar vidas”, funções que foram afetadas pela COVID-19, não por falta de desejo ou iniciativa dessa categoria, mas pelo desconhecimento de como lidar com esse novo vírus e suas repercussões na saúde da população. Apesar de possuírem, em sua maioria, um amplo capital cultural institucionalizado, esse conhecimento não foi suficiente para realizar a cura imediata e evitar a morte dos pacientes com a doença. Tal situação foi especialmente observada no início da pandemia, quando não havia vacina nem manejo eficaz para tratar essa doença até então desconhecida. Esses profissionais sofreram uma desarmonia entre o desejo de fornecer cuidados abrangentes aos pacientes com a COVID-19 e as limitações impostas pelo Sistema de Saúde nacional, afetando, inclusive, seu *habitus*. O ato de cuidar e curar faz parte do *habitus* desses profissionais, incorporado ao longo de sua trajetória de vida e carreira.

Diversos recursos internos foram utilizados por esses profissionais na tentativa de combater o vírus e prestar assistência aos doentes. Esses recursos provinham principalmente do capital cultural institucionalizado, que remete aos títulos escolares acumulados, e do capital cultural incorporado, adquirido e internalizado por meio de investimentos de longa duração, que passaram a ser parte integrante da pessoa (agente)^(11)^. O capital cultural incorporado antes da pandemia foi resgatado e aplicado pelos profissionais de saúde para lidar com a situação da pandemia em seu campo de atuação. Atuaram com altruísmo, característico da profissão.

Também emergiu a questão da exposição prolongada dos profissionais de saúde a fatores de estresse. O estresse ocupacional foi percebido no campo de atuação, com demandas que excediam sua capacidade de enfrentamento, gerando reações negativas na saúde e no bem-estar dos trabalhadores. A pandemia evidenciou uma grave vulnerabilidade dos profissionais de saúde, além da exposição contínua a fatores externos como riscos biológicos, econômicos e estruturais, bem como fatores individuais^(30,31)^.

O grupo de profissionais que atuou na linha de frente do cuidado era constituído por agentes sociais que, em sua maioria, atuavam no ambiente hospitalar. Trata-se de um espaço dinâmico e instável, onde se adquire conhecimentos por meio do contato com diferentes estruturas sociais, o que permite ao agente agir e evoluir com naturalidade, caracterizando o *habitus* profissional. O *habitus* profissional desse grupo foi constituído a partir de exposições repetidas às condições próprias do ambiente hospitalar, imprimindo neles um conjunto de disposições duráveis e transferíveis, interiorizadas a partir da necessidade de permanência e de se situar nesse universo social^(11)^.

O ambiente hospitalar durante a pandemia da COVID-19 organizou-se em um campo caracterizado por agentes dotados de um mesmo *habitus*. No campo ocupado, seja ele em um ambiente público ou privado, foram identificadas mudanças nas rotinas e imposições de poder. O poder imposto pelas instâncias federais gerou dominação no campo, em ambos os ambientes. A partir do *habitus* e do capital que cada profissional de saúde incorporou em sua trajetória social e profissional, foi possível manter-se atuante durante a pandemia da COVID-19.

Esse ambiente (espaço social), no qual os agentes se posicionavam, foi reorganizado para atender à alta demanda exigida pela pandemia. As transformações foram realizadas com o objetivo de ofertar à população o melhor possível em termos de promoção, proteção, recuperação e reabilitação da saúde, mas apresentaram repercussões em múltiplas dimensões^(32)^.

O agente inserido no campo foi produzindo estratégias que se mostraram ajustadas à situação de pandemia. Mesmo sem plena consciência, o agente (profissional de saúde) foi adquirindo um senso prático pela exposição continuada a situações semelhantes, e, com isso, foi interiorizando diferentes formas de agir^(11)^. As estruturas adquiridas no campo se tornaram capitais. Aqueles que passaram por treinamento e capacitação direcionada ao cuidado com o novo coronavírus tiveram a oportunidade de mudanças em suas ações, práticas e até mesmo na posição dentro do campo hospitalar, em virtude de um capital novo incorporado. Nota-se que a evolução da posição ocupada pelo profissional depende de seu capital e das estratégias implementadas. Foi identificado que os profissionais de saúde buscavam, em conjunto, reconhecimento e melhores posições dentro do campo hospitalar no combate à pandemia, sem que houvesse destaque de uma categoria em detrimento de outra.

Outro desafio enfrentado pelos profissionais de saúde foi a questão do cuidado infantil, especialmente porque as escolas permaneceram fechadas por muito tempo no Brasil. Muitos idosos, que faziam parte da rede de apoio dos profissionais de saúde, precisavam evitar contato. Com isso, muitos profissionais tiveram que trabalhar e, ao mesmo tempo, cuidar de seus filhos, o que tornou essa logística mais difícil e complicada^(33)^.

Os profissionais de saúde desejavam uma garantia inequívoca de que suas organizações os apoiariam, bem como às suas famílias. O desejo desses profissionais era por uma liderança visível durante esse período turbulento e pelo reconhecimento do trabalho realizado^(34)^. No entanto, os dados apontam que esse apoio não foi percebido pelos profissionais de saúde, independentemente de sua categoria profissional.

Verificaram-se relatos de suspensão de férias. Vale ressaltar que, pela Medida Provisória n.º 927/2020, de 22 de março de 2020, em seu artigo 7º, a suspensão das férias ou das licenças não remuneradas dos profissionais da área de saúde ou daqueles que desempenham funções essenciais durante o período de calamidade pública atende ao interesse público, que se sobrepõe ao interesse individual.

Também destacaram sentimentos de impotência, necessidade de políticas públicas e ações governamentais. A pandemia da COVID-19 no Brasil tornou-se um evento crítico de múltiplas proporções, cujas repercussões foram agravadas pela combinação desastrosa de um presidente caracterizado como genocida e um governo comandado pelo capital econômico^(35)^. O poder simbólico exercido pelos dirigentes responsáveis pelas decisões sobre a pandemia foi determinado pelo poder das palavras pronunciadas pelos ministros da saúde e pelo presidente da república.

Bourdieu (2004) afirma que a hierarquia simbólica reforça a hierarquia social, pela divisão entre dominantes e dominados conforme o volume e os tipos de capitais acumulados. O capital desses profissionais não tinha peso nas posições ocupadas, pois não era o capital que determinava a posição social desses agentes, mas sim a hierarquia simbólica à qual estavam submetidos^(36)^.

Na análise dos dados do estudo, não foi observado relato de relação de poder e dominação entre as categorias profissionais, o que poderia ter ocorrido, uma vez que as categorias apresentam capitais culturais e até mesmo específicos diferenciados. Talvez isso possa ser compreendido como uma necessidade de se apoiarem dentro de um contexto de pandemia. Contudo, foram verificados o poder e a hierarquia simbólica exercidos pelo Estado, que geravam uma sensação de impotência e ansiedade nos trabalhadores da saúde que estavam na linha de frente do cuidado. Os segmentos de texto expressos pelos profissionais apontavam sentimentos de preocupação e insatisfação pela falta de apoio de gestores. As principais preocupações dessa categoria incluíam a falta de apoio institucional e a postura de algumas autoridades governamentais e funcionários do Estado que subestimavam a gravidade da pandemia, fato que contribuiu para a disseminação da COVID-19 e para um sentimento de insegurança, ansiedade e falta de perspectivas de um futuro melhor^(25,37)^.

Houve uma resposta coordenada à doença causada pelo Coronavírus, com a ativação, em 22/01/2020, do Centro de Operações de Emergência em Saúde Pública (COE-COVID19), trazendo definições de casos, orientações para notificação, investigação laboratorial, isolamento e transporte, além de cuidados com o paciente na atenção primária e especializada, bem como medidas de higiene^(37)^. O SUS tinha, entre seus objetivos, o estabelecimento de mecanismos ágeis para registro dos casos, aquisição, distribuição, avaliação e monitoramento da qualidade de produtos, equipamentos e insumos relacionados às respostas à pandemia, além da implementação de diretrizes para minimizar o impacto e a disseminação da pandemia^(38)^.

Na prática, não havia uma coordenação central, o que gerava, para quem estava na linha de frente da assistência, uma sensação de descontrole. A impotência, frustração e falta de informações fidedignas relatadas não eram específicas de um campo, nem restritas ao cenário público ou privado de atuação dos agentes, tampouco a uma única categoria profissional, e sequer reservadas aos agentes com menos capital acumulado.

A pandemia levou ao esgotamento físico dos profissionais de saúde. Vários aspectos foram problematizados, com termos como “cansado”, “exausto”, “esgotado” e “sem perspectiva” se destacando, inclusive na nuvem de palavras. Pelo *habitus*, o profissional de saúde permaneceu no campo de atuação e recorreu a seu capital cultural incorporado. No entanto, a exposição prolongada ao SARS-CoV-2 e as repercussões da COVID-19, de forma ininterrupta, afetaram não apenas o aspecto emocional, mas também o físico desses profissionais.

Ao observarmos os pontos centrais destacados, identificamos que convergem em uma violência simbólica direcionada pela instância federal (dominadora) aos profissionais de saúde (dominados) que estavam na linha de frente da assistência durante a pandemia da COVID-19. As diferentes categorias da área da saúde, independentemente do *habitus* e do capital acumulado, aproximavam-se pela identificação da necessidade de uma mudança resolutiva no campo durante a pandemia, mas, devido à posição ocupada como dominados, não conseguiam transformar essa realidade.

### Limitaçõ,42 es do estudo

Houve uma maior resposta ao preenchimento dos questionários por profissionais médicos e enfermeiros, decorrente do perfil dos próprios pesquisadores, que têm uma rede de contatos formada, em sua maioria, por profissionais dessas categorias. O mesmo ocorre em relação a uma maior frequência do gênero feminino. Os pesquisadores deste trabalho atuam nas áreas da saúde da mulher e da criança, onde a presença feminina é predominante. Essa situação limita a extrapolação dos resultados para outros perfis, mas, por outro lado, permite uma análise mais precisa desse grupo, que é bastante expressivo entre os profissionais de saúde.

### Contribuições para a Área da Enfermagem

A pesquisa contribui significativamente para a enfermagem ao evidenciar os impactos da COVID-19 na saúde física e mental dos profissionais de saúde no Rio de Janeiro. Identifica sentimentos como medo e esgotamento, ressaltando a necessidade de cuidados abrangentes. A análise das relações simbólicas destaca a violência muitas vezes imperceptível. A conclusão enfatiza a importância de medidas para proteger a saúde e os direitos trabalhistas e promover o bem-estar dos profissionais de saúde.

## CONSIDERAÇÕES FINAIS

Ao reunir e interpretar as falas dos profissionais, foi possível identificar diferentes formas de violência vivenciadas por eles em seu ambiente de trabalho e no contexto que os cerca. Com a utilização do referencial teórico de Bourdieu, observou-se nos relatos dessas categorias profissionais dentro de um campo dinâmico que o *habitus*, o capital cultural e as estratégias adotadas pelos agentes foram determinantes para a persistência e sobrevivência em meio à violência simbólica vivida durante a pandemia. A violência simbólica foi exercida por meio de um poder simbólico imposto aos profissionais de saúde (dominados) de forma silenciosa, mas que permeou o campo de atuação dessa categoria. À medida que se negou suporte, condições de trabalho e recursos materiais para o enfrentamento da pandemia, exerceu-se uma violência simbólica que, para muitos, é imperceptível, mas real, gerando limitações na classe dominada. Trata-se de uma violência insensível, porém exercida essencialmente pelas vias simbólicas da comunicação e do conhecimento, com ações de submissão sobre os dominados. Estas análises expuseram que os diferentes profissionais de saúde, ao exercerem suas funções laborais, vivenciaram mazelas em forma de violência simbólica muitas vezes não declarada.
